# Cancer-derived extracellular succinate: a driver of cancer metastasis

**DOI:** 10.1186/s12929-022-00878-z

**Published:** 2022-11-07

**Authors:** Cheng-Chin Kuo, Jing-Yiing Wu, Kenneth K. Wu

**Affiliations:** 1 Institute of Cellular and System Medicine, National Health Research Institutes, 35 Keyan Road, Zhunan Town, Miaoli County 35053 Taiwan; 2 Graduate Institute of Basic Medical Science, China Medical University, Taichung, Taiwan; 3 Institute of Biotechnology, National Tsing-Hua University College of Life Science, Hsinchu, Taiwan

**Keywords:** Succinate, Succinate receptor-1, G-protein-coupled receptor 91 (GPR91), Cancer metastasis, Hereditary paraganglioma, Succinate dehydrogenase

## Abstract

Succinate is a tricarboxylic acid (TCA) cycle intermediate normally confined to the mitochondrial matrix. It is a substrate of succinate dehydrogenase (SDH). Mutation of SDH subunits (SDHD and SDHB) in hereditary tumors such as paraganglioma or reduction of SDHB expression in cancer results in matrix succinate accumulation which is transported to cytoplasma and secreted into the extracellular milieu. Excessive cytosolic succinate is known to stabilize hypoxia inducible factor-1α (HIF-1α) by inhibiting prolyl hydroxylase. Recent reports indicate that cancer-secreted succinate enhances cancer cell migration and promotes cancer metastasis by activating succinate receptor-1 (SUCNR-1)-mediated signaling and transcription pathways. Cancer-derived extracellular succinate enhances cancer cell and macrophage migration through SUCNR-1 → PI-3 K → HIF-1α pathway. Extracellular succinate induces tumor angiogenesis through SUCNR-1-mediated ERK1/2 and STAT3 activation resulting in upregulation of vascular endothelial growth factor (VEGF) expression. Succinate increases SUCNR-1 expression in cancer cells which is considered as a target for developing new anti-metastasis drugs. Furthermore, serum succinate which is elevated in cancer patients may be a theranostic biomarker for selecting patients for SUCNR-1 antagonist therapy.

## Introduction

Succinate is a tricarboxylic acid (TCA) cycle intermediate normally confined to mitochondrial matrix where it serves as a substrate for succinate dehydrogenase (SDH) and an electron donor for electron transport chain (ETC) [1]. When cells are under stressful conditions such as hypoxia, hyperglycemia and endotoxemia, TCA cycle is broken resulting in mitochondrial matrix succinate accumulation. Several mechanisms including SDH dysfunction, fumarate overproduction and/or TCA acceleration contribute to increased succinate [2, 3]. Excessive succinate leaks to cytoplasma and is secreted into extracellular space [3–5]. Cytosolic and extracellular succinate accumulation promotes cancer growth by distinct mechanisms. The intracellular succinate being structurally similar to α-keto-glutarate (α-KG) acts as a competitive inhibitor of a large number of α-KG-dependent enzymes (or 2-oxoglutarate-dependent dioxygenases, 2OGDD) which utilize α-KG as a co-substrate to catalyze diverse reactions and carry out important functional roles in protein hydroxylation, histone and DNA demethylation, collagen biosynthesis and energy metabolism [6]. Several of the α-KG-dependent enzymes including prolyl hydroxylase (PHD), TET family of 5’-methylcytosine hydroxylases, DNA and histone demethylases are dysregulated in cancer [6]. Cytosolic succinate accumulation as a result of SDH mutation was reported to inhibit α-KG-dependent histone and DNA demethylases which contributes to tumorigenesis [7]. Of note, cytosolic succinate stabilizes hypoxia inducible factor-1α (HIF-1α) by inhibiting PHD which is required for HIF-1α degradation via the ubiquitin–proteasome system [8, 9]. HIF-1α mediates transcription of genes important in glycolysis, inflammation and angiogenesis [10, 11]. On the other hand, the extracellular succinate exerts potent biological actions by activating a plasma membrane G protein-coupled receptor, i.e., succinate receptor-1 (SUCNR-1, also known as GRP91) [12]. Extracellular succinate has been shown to play an important role in enhancing inflammation, inducing tissue fibrosis and regulating renin-angiotensin and hypertension [13–16].

Succinate accumulation was detected in tumors with germline mutation of SDH and cytosolic succinate was recognized as a signal molecule to stabilize HIF-1α and alter tumor behavior. However, it was unclear whether cancer cells secrete succinate into the extracellular milieu. Nor was it known that extracellular succinate possesses biological activities to influence cancer behavior and progression. Reports from recent studies start to shed light on succinate secretion by cancer cells and the crucial role that extracellular succinate plays in promoting cancer progression especially cancer metastasis. The purpose of this review is to address the underlying mechanism of succinate accumulation in and secretion from cancer cells, actions of succinate on cancer cell migration, invasion and metastasis and the potential value of SUCNR-1 as a target for developing new anti- metastatic therapy.

### Succinate accumulation due to SDH deficiency in tumor cells

SDH is pivotal in mitochondrial metabolism and bioenergetics. It possesses catalytic activity to convert succinate to fumarate in TCA cycle and an electron transfer system for electron transport in ETC. It is a mitochondrial protein complex comprising four subunits i.e., SDHA, B, C and D and assembly factors [17, 18]. SDH subunits and assembly factors are encoded by genes in the nuclear genome [19]. Structurally, SDHC and SDHD subunits are embedded in the inner membrane and serve as the anchor for SDHB and SDHA subunits. In addition, the interface of SDHC and SDHD contains heme and ubiquinone binding sites which are essential for electron transfer. SDHB binds directly to SDHC and SDHD while SDHA binds SDHB. SDHA contains FAD binding site and catalytic site where succinate is oxidized to fumarate. Succinate donates electrons to FAD to form FADH_2_ which serve as electron donors in ETC. SDHB subunit contains three iron-sulfur clusters to facilitate electron transfer to SDHC and SDHD where ubiquinone is reduced to ubiquinol. In addition to being a TCA cycle enzyme, SDH complex functions as complex II in the ETC. Each of the four subunits is essential for SDH catalytic activity and electron transport function. Mutation or reduced expression of individual subunit disrupts SDH integrity and diminishes SDH catalytic activity leading to succinate and reactive oxygen species (ROS) accumulation and impaired oxidative phosphorylation [20, 21].

**Fig. 1:**
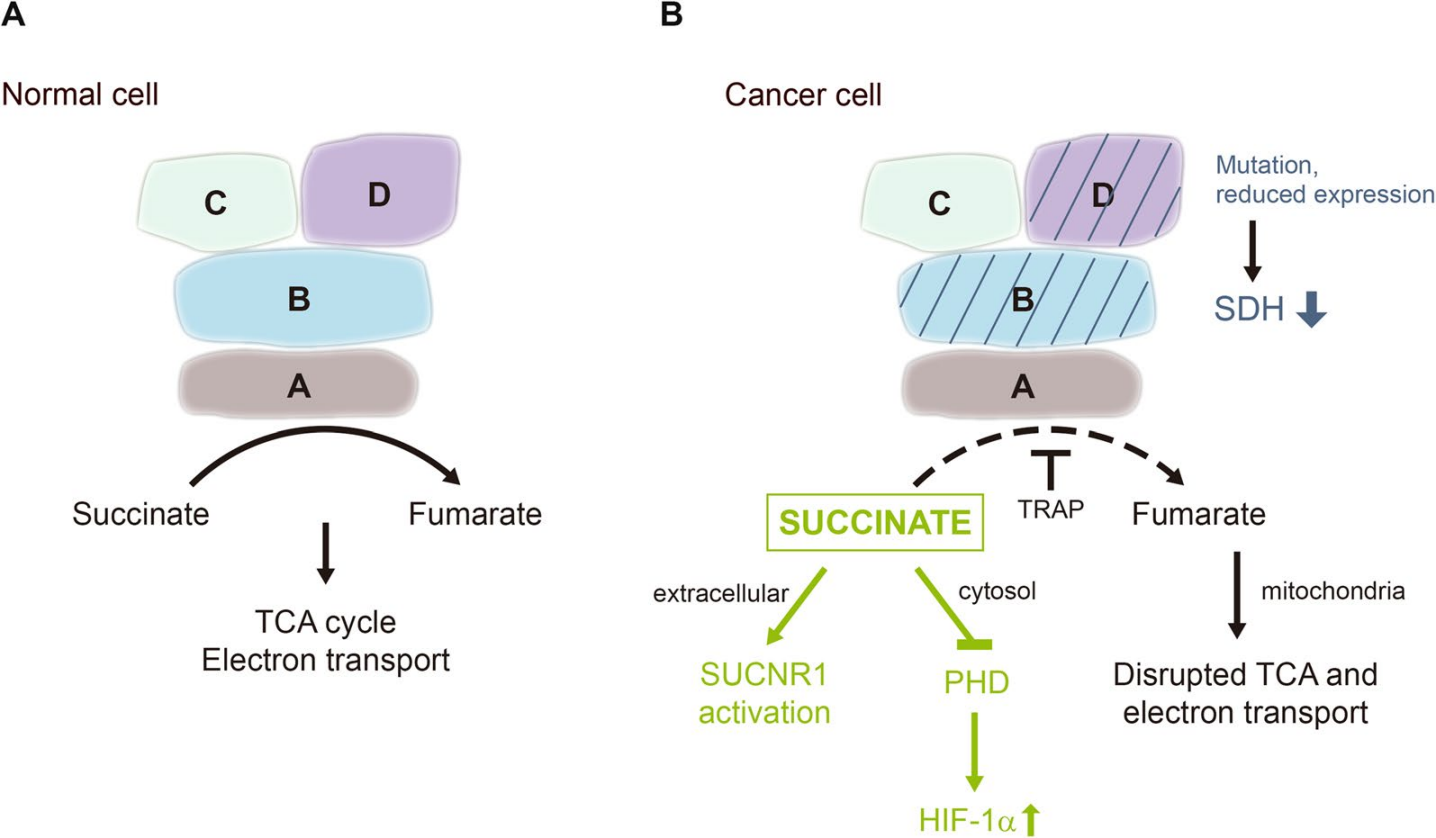
Succinate dehydrogenase (SDH) in normal and cancer cell. **A**. Schematic illustration of SDH subunits. It catalyzes conversion of succinate to fumarate in TCA cycle and electron transport in ETC. **B** Mutation or expression defect of subunit B or D results in reduced SDH activity and accumulation of succinate. TRAP inhibits SDH catalytic activity also resulting in succinate accumulation. Cytosolic succinate stabilizes HIF-1α through inhibition of PHD while extracellular succinate promotes cancer metastasis via SUCNR-1

Mammalian cell SDH activity is regulated by extrinsic factors notably TNF receptor associated protein (TRAP). Increased TRAP-1 expression suppresses SDH activity resulting in succinate accumulation. Sirt3, a member of the Sirtuin family is a mitochondrial deacetylase which regulates lysine acetylation of several mitochondrial proteins including SDHA subunit [22]. Sirt3 binds to SDHA and increases SDH electron transfer and catalytic activity. Sirt3 is considered to be a tumor suppressor [23], and modulation of its expression was reported to enhance the effect of sorafenib on hepatocellular carcinoma [24]. Sirt3 suppresses cancer progression and metastasis by controlling acetylation of several mitochondrial proteins including superoxide dismutase II (SOD II) via which it decreases ROS [25]. It remains to be investigated whether Sirt3 regulates cancer cell migration and metastasis through controlling succinate accumulation and/or secretion. Succinate accumulation in tumor cells due to SDH mutation, defective expression and inhibition is summarized in Fig. 1.

#### Succinate accumulation due to SDH mutations in hereditary and sporadic tumors

Germline mutation of SDH was first reported in hereditary paraganglioma [26], and subsequently confirmed in diverse hereditary and sporadic tumors including hereditary and sporadic pheochromocytoma, gastrointestinal stromal tumors and familial renal cell carcinoma [27–30]. Germline mutations of SDHB and SDHD subunits which are commonly detected in hereditary and sporadic tumors result in collapse of SDH catalytic activity and complex II function leading to succinate accumulation and excessive ROS generation [20, 21]. Of note, SDHB mutations are associated with malignant and metastatic tumors such as malignant pheochromocytoma, renal cell carcinoma and neuroendocrine tumors [31, 32].

#### Succinate accumulation due to reduced SDH expression in cancer cells

In addition to SDH subunit mutations which occur in hereditary tumors and sporadic cancers, loss of SDH function may be the result of loss of heterozygosity [33] or reduction of SDHB or SDHD subunit expression in diverse cancers including colorectal, gastric, hepatocellular, ovarian and clear cell renal cell carcinoma (ccRCC) [34–37]. The mechanism by which SDHB or SDHD expression is reduced in cancer cells is not entirely clear. A recent report on ccRCC suggests that reduced SDHD expression is attributable to degradation of SDHD transcripts mediated by microRNA-210 (miR210) upregulation [34]. miR210 induces SDHD degradation by binding to the 3’-untranslated region of SDHD mRNA. Reduced SDH catalytic activity leads to accumulation of succinate in renal tissues, increased cancer cell invasion and poor prognosis [34]. Reduced SDHB expression in hepatocellular cancer (HCC) is also associated with poor prognosis [35]. Several reports have indicated that SDHB plays an important role in controlling cancer metastasis. Silencing of SDHB in cancer cells with selective siRNA results in phenotypic changes characterized by enhanced migration and invasion, epithelial mesenchymal transition (EMT) and metabolic switch to glycolysis [34, 36, 37]. Aspura et al. reported that knockdown of SDHB results in reduced SDH catalytic activity accompanied by increased cell proliferation, EMT and metabolic switch [36]. Chen et al. reported that overexpression of SDHB in ovarian cancer cells reduces cell proliferation, migration and invasion [37]. By contrast, Cervera et al. reported that silencing of SDHB in HCC and gastric carcinoma cells reduces cell proliferation despite loss of SDH catalytic activity [38]. The reason for the discrepancy has not been resolved. One possible explanation is differences in cell types and experimental conditions. Nevertheless, the reported findings support the notion that reduction of SDH activity in cancer cells has a profound effect on cancer phenotype characterized by increasing cancer cell migration, invasion and cancer metastasis.

#### Succinate accumulation due to inhibition of SDH activity by TRAP-1

TRAP-1 is a mitochondrial chaperone which forms complex with heat shock protein 90 (HSP90) in the mitochondrial matrix [39, 40]. TRAP-1/HSP90 play an important role in mitochondrial quality control, redox balance, bioenergetics and membrane permeability. TRAP-1 is overexpressed in diverse types of cancers and was reported to accelerate prostate cancer development [41]. One of the mechanisms by which TRAP-1 promotes cancer growth is through binding to SDH thereby inhibiting SDH catalytic activities (Fig. 1) and disrupting electron transfer [42], leading to succinate and ROS accumulation.

### Succinate accumulation drives cancer growth and metastasis

**Fig. 2:**
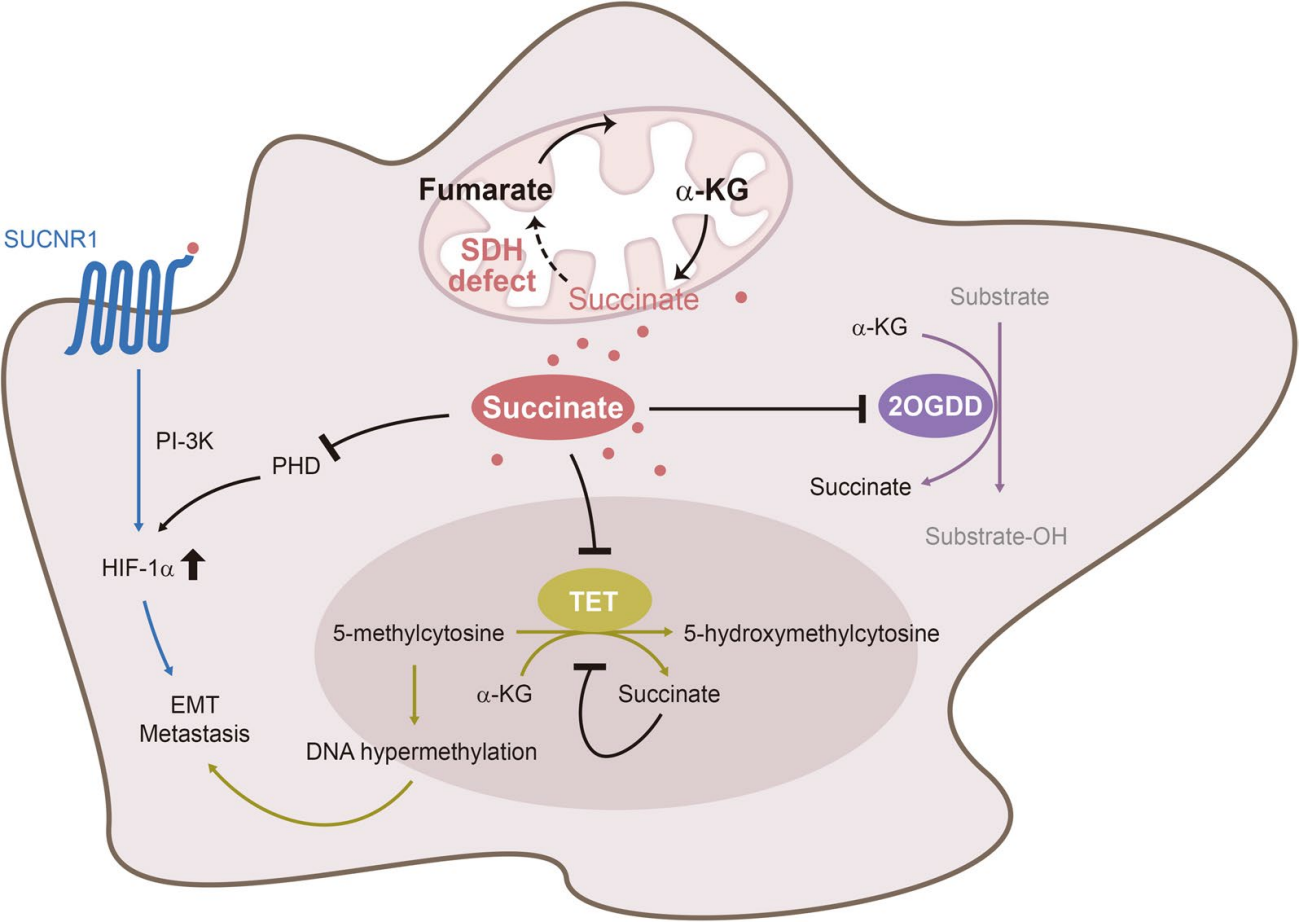
Succinate promotes cancer cell growth by inhibiting 2OGDD and through ligating SUCNR-1. Succinate accumulation due to SDH defect leads to increased cytosolic succinate and secretion of succinate. Cytosolic succinate exerts its effect by inhibiting a large number of 2-oxoglutarate-dependent dioxygenases (2OGDD or α-keto glutarate-dependent enzymes) such as prolyl hydroxylase (PHD) and TET (Ten to Eleven Translocation). Inhibition of PHD results in HIF-1α stabilization while inhibition of TET leads to DNA hypermethylation. Extracellular succinate interacts with succinate receptor-1 (SUCNR-1) which signals via PI-3 K to increase HIF-1α expression

Earlier studies recognized that loss-of-function mutation of SDHD in paraganglioma was associated with elevated HIF-1α which led to the proposal that SDH mutation results in activating hypoxia response pathway [20, 43–45]. It was subsequently discovered that HIF-1α elevation is attributed to cytosolic succinate accumulation [6]. Under normoxic conditions, HIF-1α is rapidly degraded by ubiquitin-proteosome system. A pre-requisite for degradation is hydroxylation of HIF-1α prolyl residues catalyzed by PHD [7–9]. Succinate inhibits PHD activity thereby blocking HIF-1α degradation [6] (Fig. 2). HIF-1α is a pleiotropic transcription activator, mediating transcription of tumor promoting genes including pro-metastatic genes [10, 11]. Extracellular succinate augments cancer growth and metastasis through SUCNR-1 mediated signaling pathways and transcription programs. It is of interests to note that cancer cells secrete the glycolytic end product, lactic acid which was shown to be an active extracellular signaling molecule in promoting cancer growth [46, 47]. Wu et al. analyzed metabolites in the conditioned medium (CM) of lung (A549, LLC), prostate (PC3), breast (MCF7), and colon (HT29) cancer cells by liquid chromatography-mass spectrometry (LC–MS). They reported detection of abundant succinate in the CM of all the cancer cells tested [48]. Lactate was also detected but was less abundant. On the other hand, succinate and lactate were undetectable in the CM of unstimulated macrophages [48]. Mu et al. reported detection of succinate in the CM of gastric cancer cells by colorimetry [49]. Cells stimulated by stress signals such as ischemia–reperfusion (I/R) or lipopolysaccharide (LPS) release succinate into the excellular milieu [3, 15] through monocarboxylate transporter-1 (MCT-1) [50, 51]. MCT-1 expression is upregulated in several types of cancer cells which is considered to be involved in tumor growth [52, 53]. Cancer cell may secrete succinate via this membrane transporter. Succinate secreted from LPS-stimulated macrophages and I/R-injured cells amplifies inflammatory responses and induces tissue damage and fibrosis [14, 15, 54–56]. Cancer-derived succinate, on the other hand, promotes cancer metastasis by driving cancer cell migration and invasion, inducing epithelial mesenchymal transition (EMT) and angiogenesis, which will be described below.

#### Cancer cell-derived succinate enhances cell migration and drives EMT by interaction with SUCNR-1

Succinate released from human lung (A549) or murine lung (LLC) cancer cells enhances cancer cell migration and induces cancer cell EMT [48]. By contrast, cancer-derived succinate does not influence cancer cell viability or proliferation. The specific effect of succinate on cancer cell migration and EMT results in increased cancer metastasis as demonstrated in a syngeneic murine tumor model in which C57BL/6 J mice were implanted with LLC murine lung cancer cells subcutaneously. Administration of succinate in vivo increases cancer metastasis to lungs and other organs, but has no effect on tumor volume [48].

Succinate drives cancer cell migration and enhances cancer metastasis by interaction with SUCNR-1 on cancer cell surface. Cancer cell-derived succinate activates SUCNR-1 resulting in ERK1/2 activation, prostaglandin E2 production and increased intracellular calcium [57, 58]. Furthermore, succinate activates p38 MAPK, Akt and AMPK in lung cancer cells, suggesting that succinate activates multiple signaling pathways via SUCNR-1 interaction. Of note, only PI-3 K/Akt inhibition abrogates succinate-induced cancer cell migration [48]. Interestingly, extracellular succinate induces HIF upregulation via membrane receptor-PI-3 K signaling (Fig. 2). HIF-1α and -2α overexpression promotes cancer cell invasion and drives EMT by a number of biochemical and genetic processes including Twist transcription, Snail nuclear localization and the consequent loss of E-cadherin and increase in mesenchymal markers [59–61]. Experimental results from murine xenograft tumor model support the crucial role of HIF-1α in cancer metastasis. Implantation of HIF-1α silenced A549 cells into nude mice results in reduced lung metastatic nodules accompanied by reversal of E-cadherin and vimentin expression in tumor cells [48].

**Fig. 3:**
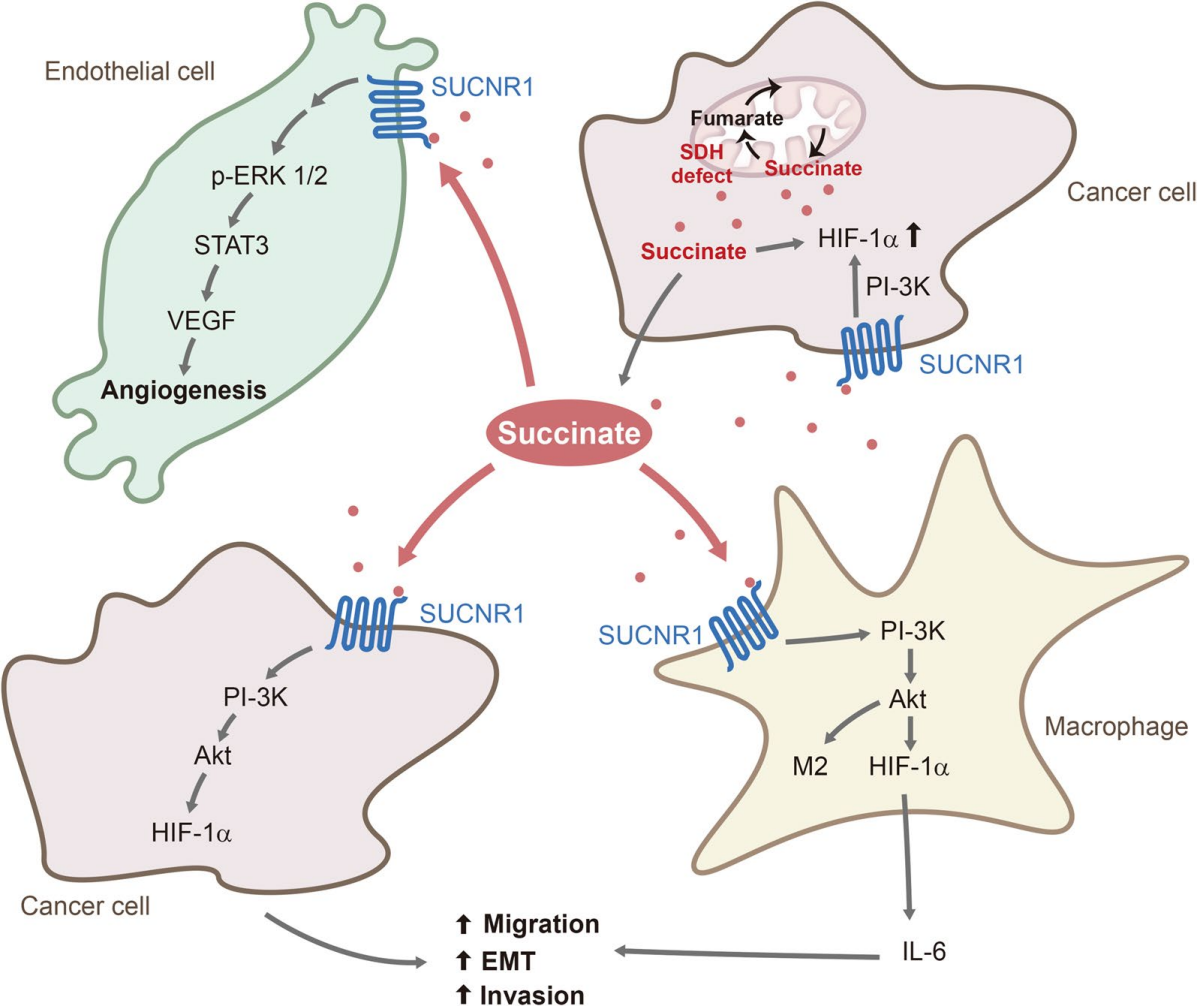
Cancer cell-derived succinate acts on macrophages, endothelial cells and cancer cells to drive cancer metastasis

Succinate accumulation in ccRC with decreased SDHD or SDHB expression was reported to enhance cancer cell invasion and metastasis by increasing DNA 5-methylcytosine (5mC) and suppressing 5-hydroxymethylcytosine (5-hmC) through inhibition of TET-2, resulting in global DNA hypermethylation [34]. TET-2 (Ten to Eleven Translocation-2) is an α-KG-dependent enzyme which requires α-KG as a co-substrate to catalyze conversion of 5mC to 5-hmC (Fig. 2). DNA hypermethylation contributes to cancer invasiveness and EMT by altering binding of transcriptional activators to promoters of genes regulating migration and EMT. For example, HIF-1α binding to the target genes is influenced by DNA methylation [62]. The reported findings suggest that succinate acts in an autocrine and paracrine manners to drive cancer metastasis via the SUCNR-1 → PI-3K → Akt → HIF-1α signaling pathway (Fig. 3).

#### Succinate activates endothelial cells and induces angiogenesis via SUCNR-1 → ERK → VEGF pathway

Cancer metastasis is further enhanced by succinate-mediated tumor angiogenesis which is essential for tumor growth and metastasis [63, 64]. For example, gastric cancer-derived extracellular succinate induces endothelial cell proliferation and angiogenesis by interacting with SUCNR-1 which transmits signals for angiogenesis via STAT3 and ERK1/2 resulting in upregulation of vascular endothelial growth factor (VEGF) [49] (Fig. 3). Tumor angiogenesis induced by gastric cancer-derived succinate is independent of HIF-1α [49]. On the other hand, extracellular succinate induces retinal angiogenesis in retinopathy and synovial angiogenesis in arthritis through signaling and transcriptional pathways depending on HIF-1α [65]. HIF-1α has been incriminated as an important factor for inducing pathological angiogenesis [65, 66]. As cellular and molecular factors participating in cancer metastasis are many and complex, current knowledge on the mechanisms by which succinate promotes cancer metastasis is incomplete. Further investigations are required.

### Extracellular succinate promotes macrophage migration and M2 polarization via SUNCR-1 → PI-3 K/Akt → HIF-1α signaling pathway

Cancer cell-derived extracellular succinate not only drives cancer cell migration but also exerts great influence on stromal cells such as macrophages and endothelial cells in the tumor microenvironment (Fig. 3). Succinate was reported to polarize macrophages and induce endothelial cell tube formation by distinct signaling pathways. It converts naïve macrophages into tumor associated macrophages (TAM) through a receptor-mediated signaling pathway similar to that for driving cancer cell migration and EMT [48]. It induces TAM polarization by activating macrophage membrane SUCNR-1 receptor and the downstream PI-3 K/Akt → HIF-1α signaling pathway [48]. Furthermore, it drives macrophage migration via the SUCNR-1 signaling pathway. Succinate-induced macrophage polarization augments cancer cell migration by secreting pro-migratory cytokines such as interleukin-6 (IL-6) (Fig. 3).

Extracellular succinate also targets M2 macrophages and activate M2 macrophage gene transcription via SUCNR1 to create a hyperpolarized M2 macrophage environment [67]. SUCNR-1 is coupled with Gi and/or Gq proteins depending on cell types [68]. Succinate induces hyperpolarized M2 macrophages via SUCNR-1 → Gq pathway [67]. In addition, inhibition of SDH was reported to impair T cell activation and function [69]. It is likely that cancer-derived extracellular succinate is a major player in the tumor microenvironment for immunosuppression and cancer cell immune evasion.

It is to be noted that inflammatory stimuli activate macrophages and induce macrophage secretion of succinate. Macrophage-derived excellular succinate aggravates inflammatory responses and exacerbates inflammatory disorders such as rheumatoid arthritis [15]. Macrophage migration in response to extracellular succinate is crucial for mediating inflammation in adipose tissues and aggravating obesity-induced diabetes [56]. By contrast, macrophage-derived extracellular succinate may activate SUCNR-1 on neural stem cells leading them to release anti-inflammatory factors [70]. Thus, extracellular succinate regulates inflammation in a complex manner depending on cell types and contexts. It remains to be investigated to what extent succinate in tumor microenvironment contributes to inflammation and inflammation-mediated cancer metastasis.

### Extracellular succinate promotes cell migration through induction of mitochondrial fission

**Fig. 4:**
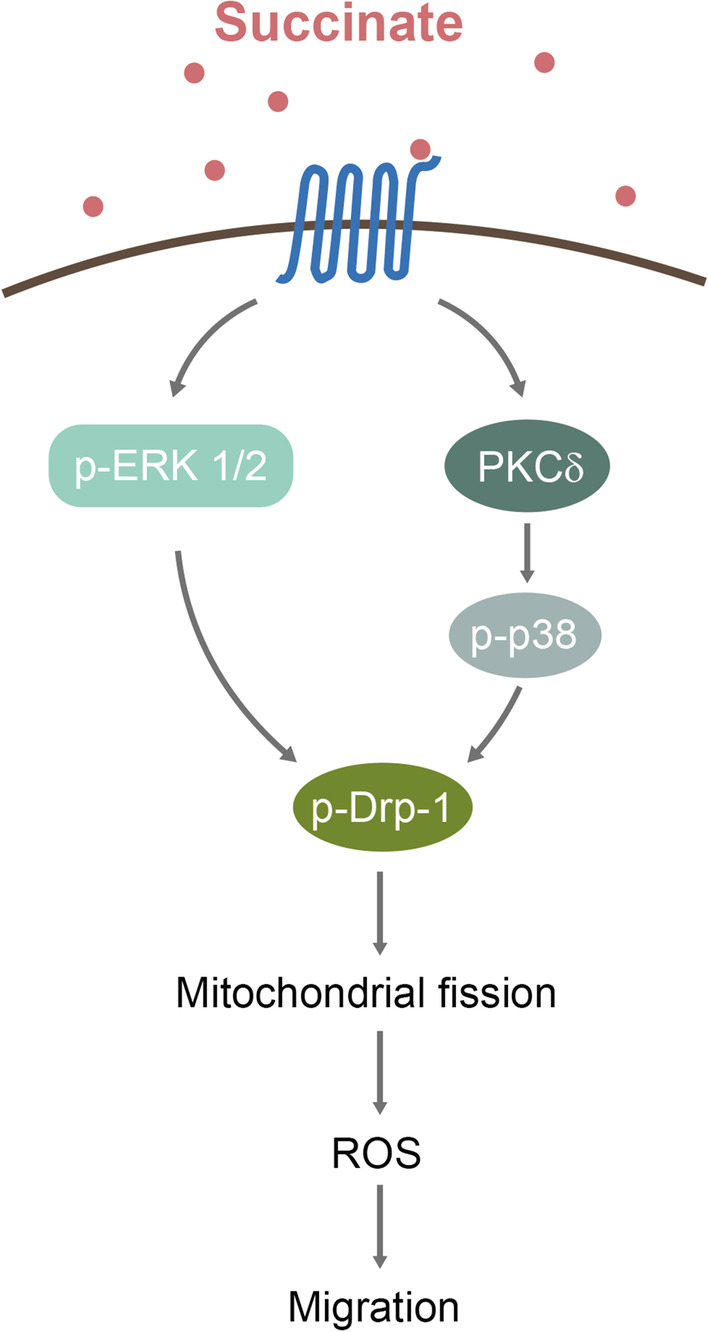
Schematic illustration of the signaling pathway via which succinate phosphorylate Drp-1 and induces mitochondrial fission. ROS generated drive cancer cell migration

Normal cells undergo constant mitochondrial fusion and fission to maintain mitochondrial dynamic balance which is vital for mitochondrial metabolism and function [71, 72]. Cancer cells exhibit an imbalanced mitochondrial dynamics with excessive fission and fragmented mitochondria [73, 74]. Several reports reveal that mitochondrial fission promotes cancer cell migration and invasion and contributes to metastasis of diverse cancers including breast, hepatocellular and thyroid cancer [75–78]. Mitochondrial fission and fragmentation in cancer cells is mediated by increased fission proteins including Drp-1, Fis-1 and/or MID49 and decreased fusion protein Mfn-1 [79]. Although extracellular succinate has not been directly linked to induction of mitochondrial fragmentation in cancer cells, a report on human mesenchymal stromal cells suggests that succinate promotes cell migration by a SUCNR-1-mediated Drp-1 phosphorylation [80]. Succinate activates SUCNR-1 coupled Gq and its downstream atypical protein kinase C, PKCδ, which in turn phosphorylates p38 MAPK (Fig. 4). p38 MAPK phosphorylates Drp-1 and induces Drp-1 translocation to mitochondrial outer membrane where it interacts with receptors such as Fis-1 and initiates mitochondrial division. Of note, it was reported that succinate induces mitochondrial fission in cardiomyocytes via SUCNR-1 → PKCδ and ERK1/2 pathway [81].

Mitochondrial fragmentation is associated with metabolic reprogramming resulting in enhanced glycolysis, and reduced oxidative phosphorylation [72]. It triggers ROS generation and membrane depolarization [82–84]. ROS play an important role in promoting cancer cell migration and invasion [85]. Furthermore, ROS drive EMT and matrix degradation [86–88]. ROS induce mitochondrial fragmentation, creating a vicious cycle [83]. Mitochondrial fission/ROS vicious cycle may be crucial in perpetuating succinate-mediated cancer cell migration and cancer metastasis [88] (Fig. 4).

### Succinate receptor is a potential target for controlling cancer metastasis

He et al. made a breakthrough discovery when they reported that succinate is a physiological ligand of an orphan G-protein coupled receptor 91 (GPR91) and linked the receptor activation to renin-angiotensin system and hypertension [12]. Toma et al. confirmed that GRP91 is succinate receptor and noted that high glucose induces extracellular succinate accumulation in kidneys which mediates renin release by activating GRP91 signaling cascade [89]. GRP91 was subsequently renamed SUCNR-1. SUCNR-1 shares with purinergic receptor structural characteristics and was initially thought to be a receptor for purinergic ligands [90, 91]. SCUNR-1 is coupled to Gi and/or Gq depending on cell types [53, 88]. A canonical downstream signal of SUCNR-1 activation is ERK1/2 activation and Ca^2+^ mobilization [92–94]. Succinate-ligated SUCNR-1 may transmit signals via non-canonical signaling pathways such as PI-3 K [48]. SUCNR-1 signaling is complex and variable depending on stimulation context, succinate concentration and cell types. How are G-proteins and its downstream signaling pathways determined and regulated is poorly understood and requires further investigation. Nevertheless, numerous reports provide evidence that succinate- activated SUCNR-1 mediates diverse pathophysiological conditions [95–99]. A high level of SUCNR-1 is expressed on cancer cells and its silencing by shRNA (short hairpin RNA) results in reducing cancer cell migration and invasion [48]. Furthermore, knockdown of SUCNR-1 in human gastric or pancreatic cancer cells restores mitochondrial function [100]. Subcutaneous implantation of lung cancer cells transfected with SUCNR-1 shRNA into a murine xenograft tumor model results in local tumor growth comparable to that from implantation of lung cancer cells transfected with control vector. However, metastatic lung nodules are significantly reduced in the SCUNR-1 shRNA group [48]. These results suggest that succinate-SUCNR-1 signaling selectively promotes cancer cell migration and cancer metastasis.

SUCNR-1 expression is increased in human SDH-mutated tumors and several common cancers, which is associated with a high risk of metastasis and a high risk of recurrence following surgery [101, 102]. It has been reported that cancer cell SUCNR-1 expression is upregulated by extracellular succinate or SDH subunit silencing, indicative of a feedback regulation [48]. SUCNR-1 was thus considered to be a target for treating SDH-mutated paraganglioma [102]. Small molecule SUCNR-1 inhibitors have been chemically synthesized [67, 103] but drug development has been slow due to concerns of adverse effects as succinate-SUCNR-1 was reported to play important physiological roles such as thermogenesis [104] and skeletal muscle adaptation to exercise [105]. Additional investigations are needed to elucidate the mechanism by which SUCNR-1 is upregulated in cancer cells and to evaluate the effects of suppressing SUCNR-1 upregulation on cancer metastasis.

### Elevated serum succinate is a potential biomarker of cancer progression

Hobert et al. were the first to detect an association between SDH mutation and elevation of serum succinate [106]. However, due to small number of patients, the reported association was uncertain and requires confirmation. Wu et al. provided experimental data to show in a syngeneic murine model that tumor growth was associated with elevation of serum succinate [48]. They further reported that serum succinate level is increased in patients with non-small cell lung carcinoma and that serum succinate has the potential to be a biomarker of lung cancer [48]. A recent report reveals that serum succinate is a biomarker of human head and neck squamous carcinoma [107]. Taken together, these findings suggest that cancer cell secrete succinate into circulating blood to raise the blood level of succinate. The biological implications of elevated serum succinate on endothelial dysfunction and cancer cell extravasation are unclear and require further investigation. Preliminary results from animal experiments suggest that elevated blood succinate promotes cancer metastasis, which may serve as a biomarker of cancer progression. It will be important to determine whether serum succinate is a theranostic biomarker for selecting patients for anti-SUCNR-1 therapy.

## Conclusion

Succinate is normally located in mitochondrial matrix serving as an intermediate metabolite of TCA cycle. Cancer cells often present with reduced expression of SDH subunits notably SDHB or SDHD resulting in succinate accumulation. Succinate accumulation may also be due to SDH inhibition by TRAP-1 which is often overexpressed in cancer. Succinate accumulated in matrix may leak to the cytosol, where it promotes cancer growth by stabilizing HIF-1α. Excessive succinate in cytoplasm is secreted into the extracellular milieu where it promotes cancer cell migration and cancer metastasis. Extracellular succinate acts in an autocrine and paracrine manner to enhance cancer cell migration by activating a specific G-protein coupled receptor, SUCNR-1 which signals via ERK1/2 and PI-3 K/Akt. Extracellular succinate may also enhance macrophage, endothelial cell and mesenchymal stromal cell migration by activating SUCNR-1 mediated signaling pathways. Cancer cell-derived succinate induces EMT, tumor angiogenesis and matrix metaloprotinase expression via SUCNR-1-mediated signaling and transcriptional pathways as well as epigenetic modification such as hypermethylation. Findings from murine xenograft tumor models support a critical role for succinate/SUCNR-1 in driving cancer metastasis. It is of interests that extracellular succinate induces excessive mitochondrial fission and fragmentation in cells under stresses such as ischemia–reperfusion and lipopolysaccharide via SUCNR-1. Given that mitochondrial fragmentation in cancer cells is associated with increased migration and metastasis, it is possible that succinate/SUCNR-1 → mitochondrial fragmentation pathway may provide additional force to drive cancer metastasis.

Extracellular succinate mediates diverse actions via SUCNR-1. SUCNR-1 is a reasonable target for controlling metastasis. However, targeting SUCNR-1 may be confronted by perturbation of normal physiological functions mediated by SUCNR-1. Further studies are needed to develop therapeutic agents based on differential inhibition of SUCNR-1 on cancer metastasis vs. normal cellular physiological functions.

Cancer cell-secreted succinate contributes to elevation of blood succinate levels. Serum succinate was reported to be a potential biomarker of lung cancer. It is important to determine whether serum succinate is a theranostic biomarker for selecting cancer patients for anti-SUCNR-1 drug therapy.

## Data Availability

Not applicable.

## Abbreviations

TCA: Tricarboxylic acid
SDH: Succinate dehydrogenase
ETC: Electron transport chain
α-KG: α-Keto-glutarate
2OGDD: 2-Oxoglutarate-dependent dioxygenases
PHD: Prolyl hydroxylase
HIF-1α: Hypoxia inducible factor-1α
SUCNR-1: Succinate receptor-1
ROS: Reactive oxygen species
TRAP: TNF receptor associated protein
SOD II: Superoxide dismutase II
ccRCC: Clear cell renal cell carcinoma
miR210: MicroRNA-210
HCC: Hepatocellular cancer
EMT: Epithelial mesenchymal transition
HSP90: Heat shock protein 90
CM: Conditioned medium
LC–MS: Liquid chromatography-mass spectrometry
I/R: Ischemia–reperfusion
LPS: Lipopolysaccharide
MCT-1: Monocarboxylate transporters-1
EMT: Epithelial mesenchymal transition
5mC: 5-Methylcytosine
5-hmC: 5-Hydroxymethylcytosine
VEGF: Vascular endothelial growth factor
TAM: Tumor associated macrophages
IL-6: Interleukin-6
GPR91: G-protein coupled receptor 91
Drp-1: Dynamin-related protein-1
